# Supplementary material for: RNA-Seq Analysis Reveals Expression Regulatory Divergence of W-Linked Genes between Two Contrasting Chicken Breeds
Source: Animals (Basel). 2022 May 9;12(9):1218. doi: 10.3390/ani12091218 (PMC9103786; doi:10.3390/ani12091218)
Supplement: Supplementary file 1 [file animals-12-01218-s001.zip › animals-1624411 - Supplementary.pdf]

Table S1. Statistical results of regulatory divergence of DEGs.

Gene ID	Tissue [a]	FC [b]	Regulatory Diversity [c]
LOC121108191	B	1.25	Cd(1),Con(2)
LOC121108223	B / L	1.31 / 1.36	Cd(1),Con(2) / Td(1),Con(2)
LOC121108182	B / M	3.78 / 0.27	Ta(1),Ta(2) / Td(1),Tu(2)
SKA1	B / L	0.23 / 0.41	Ta(1),Ta(2) / Ta(1),Td(2)
LOC100859467	B	1,38	Cd(1),Td(2)
LOC100859602	B	1.33	Cd(1),Td(2)
LOC121108181	B / M	1.50 / 1.44	Cd(1),Td(2) /Cd(1),To(2)
LOC121108196	B / M	1.50 / 1.49	Cd(1),Td(2) /Tu(1),Td(2)
LOC121108216	B	1.97	Cd(1),Ta(2)
LOC374195	B/ M	1.48 / 1.34	Cd(1),Td(2) /Tu(1),Td(2)
LOC769000	B	1.30	Cd(1),Td(2)
LOC107055446	B	1.38	Ta(1),Td(2)
LOC426615	B / L	1.33 / 0.77	Cd(1),Td(2) / Cd(1),Td(2)
NEDD4L	B	1.30	Cd(1),To(2)
SMAD7B	B / M	1.31 / 1.35	Con(1),Td(2) / Td(1),Td(2)
LOC101750268	B / L	2.41 / 14.47	Ta(1),Ta(2) / Ta(1),To(2)
LOC112531967	B / L /M	1.34 / 1.44 / 1.61	Tu(1),Tu(2) / Td(1),Con(2) /Tu(1),Tu(2)
LOC121108203	L	1.35	Cd(1),Cd(2)
LOC121108199	L / M	1.86 / 1.25	Ta(1),Cd(2) / To(1),Cd(2)
LOC121108229	L / M	2.39 / 0.63	Ta(1),Cd(2) / Td(1),Cd(2)
LOC121108197	L / M	1.60 / 1.29	Td(1),Cd(2) / Cd(1),Con(2)
LOC121108201	L / M	1.59 / 1.79	Td(1),Cd(2) / Cd(1),Ta(2)
LOC121108189	L / M	1.29 / 1.80	Cd(1),Cd(2) / Cd(1),Ta(2)
HINTW	L	1.39	Ta(1),Cd(2)
LOC121108216	L	1.27	Td(1),Con(2)
LOC121108223	L	1.36	Td(1),Con(2)
LOC121108202	L / M	1.29 / 1.69	Cd(1),Ta(2) / To(1),Cd(2)
UBAP2	L	1.36	Cd(1),Td(2)
ATP5F1AW	L / M	1.67 / 2.83	Cd(1),Td(2) / Cd(1),Cd(2)
LOC121108204	L / M	1.61 / 1.81	Cd(1),Td(2) / Cd(1),Td(2)
LOC107055485	L / M	1.86 / 0.75	To(1),Td(2) / To(1),Cd(2)
LOC107049046	L	1.41	Cd(1),To(2)
LOC121108182	L	10.43	Ta(1),To(2)
SPIN1L	L / M	0.68 / 1.37	Ta(1),To(2) / Td(1),Tu(2)
LOC431003	M	0.77	Cd(1),Cd(2)
UBE2R2L	M	1.77	Cd(1),Ta(2)
LOC430766	M	1.31	Cd(1),Td(2)
LOC427025	M	1.32	Cd(1),Td(2)
LOC121108200	M	1.39	Cd(1),Td(2)
LOC107049459	M	1.77	Ta(1),Ta(2)
LOC100859467	M	1.29	Td(1),Td(2)
LOC121108227	M	1.55	Td(1),Ta(2)
LOC112530503	M	1.58	Td(1),Td(2)
LOC121108221	M	2.51	To(1),Ta(2)
LOC107055439	M	1.48	To(1),To(2)
LOC121108224	M	0.75	Tu(1),Ta(2)
LOC121108213	M	1.30	Tu(1),Con(2)
LOC107055444	M	1.32	Tu(1),Td(2)

LOC121108220	M	0.43	Td(1),Cd(2)
MBD2	M	0.61	Tu(1),To(2)

[a] Tissue=B(Brain), (M)Muscle, L(Liver); [b] FC= Paternal Expression ratio: TPMCor/TPMWL; [c] Regulatory diversity: Cd=Cis(dominant); Td=Trans(dominant); Ta=Trans(additive); To= Trans(over-dominant); Tu=Trans(under-dominant); Con=Conserved. (1),(2): Group 1, Group 2.
